# Glucagon-like Peptide 2 Concentrations Vary in Zambian Children During Diarrhoea, in Malnutrition and Seasonally

**DOI:** 10.1097/MPG.0000000000002633

**Published:** 2020-04

**Authors:** Ellen C. Besa, Kanta Chandwe, Rosemary Banda, Likando Munalula, Lydia Kalomo, Beatrice Amadi, Paul Kelly

**Affiliations:** * Tropical Gastroenterology and Nutrition group, University of Zambia School of Medicine, London, UK; † Children’s Hospital, University Teaching Hospitals, Lusaka, Zambia, London, UK; ‡ Blizzard Institute, Queen Mary University of London, London, UK; § London School of Hygiene and Tropical Medicine, London, UK

**Keywords:** diarrhoea, glucagon-like peptide 2

## Abstract

**Objectives:**

Glucagon-like peptide 2 (GLP-2) is a 33 amino acid peptide hormone released from enteroendocrine L-cells following nutrient ingestion. It has been shown to exert trophic effects on the gut. We set out to measure GLP-2 concentrations in blood in children with diarrhoea and malnutrition.

**Methods:**

GLP-2 levels were measured in blood samples collected from 5 different groups of children (n = 324) at different time points: those with acute diarrhoea, during illness and 3 weeks after recovery; persistent diarrhoea and severe acute malnutrition; controls contemporaneous for diarrhoea; stunted children from the community; and controls contemporaneous for the stunted children. Stool biomarkers and pathogen analysis were carried out on the children with stunting.

**Results:**

GLP-2 concentrations were higher during acute diarrhoea (median 3.1 ng/mL, interquartile range 2.1, 4.4) than on recovery (median 1.8, interquartile range 1.4, 3.1; *P* = 0.001), but were not elevated in children with persistent diarrhoea and severe acute malnutrition. In stunted children, there was a progressive decline in GLP-2 levels from 3.2 ng/mL (1.9, 4.9) to 1.0 (0.0, 2.0; *P* < 0.001) as the children became more stunted. Measures of seasonality (rainfall, temperature, Food Price Index, and Shiga toxin-producing *Escherichia coli*) were found to be significantly associated with GLP-2 concentrations in multivariable analysis. We also found a correlation between stool inflammatory biomarkers and GLP-2.

**Conclusions:**

In diarrhoea, GLP-2 levels increased in acute but not persistent diarrhoea. Malnutrition was associated with reduced concentrations. GLP-2 displayed seasonal variation consistent with variations in nutrient availability.

**What Is Known**

- Diarrhoeal disease in children is still a major cause of morbidity and mortality globally.
- Highest rates of diarrhoea-related mortality occur among malnourished children.
- Glucagon-like peptide 2 is a hormone, which can be used to improve intestinal adaptation and repair.

**What Is New**

- Glucagon-like peptide 2 levels in blood rose during acute diarrhoea but not persistent diarrhoea, in which levels were similar to healthy controls.
- Glucagon-like peptide 2 levels fell progressively in children with stunting.
- Glucagon-like peptide 2 levels exhibited a seasonal pattern, with evidence of an influence of nutrient availability.

Diarrhoeal disease and malnutrition continue to contribute significantly to global childhood morbidity and mortality. Diarrhoeal disease is the second leading cause of death in children younger than 5 years, killing around 525,000 children yearly (1). In the period 2000 to 2015, it accounted for 8.6% of the total deaths in children aged 1 to 59 months (2). Treatment with oral rehydration solutions, intravenous fluids, and zinc has led to a reduction in mortality (3), but sub-Saharan Africa and Southern Asia continue to have the highest numbers of under-5 deaths despite efforts to try and reduce the disease burden (4). The majority of diarrhoea cases are of acute onset, but 3 - 20% of these cases progress to persistent diarrhoea (PD) which contributes disproportionately to morbidity and mortality (5–7). Diarrhoeal illness is also known to affect ponderal and linear growth, especially in cases of recurrent illness (8). The problem of diarrhoea is further compounded by undernutrition, which is the largest single underlying cause of death among children younger than 5 years (9). There is a vicious malnutrition-infection cycle as malnutrition lowers immune function, increasing susceptibility to infections which then contribute to undernutrition by reducing energy intake, as well as nutrient loss and malabsorption (10). Undernutrition may also be exacerbated by the poor living conditions which make one more prone to infections and disease. Children living under unsanitary conditions tend to have a generalized disturbance of small intestinal structure and function described as enteropathy. It has been implicated in growth failure through different mechanisms such as heightened permeability, gut inflammation, bacterial translocation, and nutrient malabsorption (8). Severe enteropathy has been observed in children with severe acute malnutrition (SAM) and PD (11,12) contributing to reduced surface area available for nutrient absorption and impaired barrier function (13).

Glucagon-like peptide-2 (GLP-2) is a 33-amino acid peptide growth factor that is secreted in the lower gastrointestinal tract following nutrient ingestion, particularly carbohydrates and lipids (14,15). It was first described as “enteroglucagon” (16) in a patient with a renal tumour which induced intestinal hypertrophy. GLP-2 is generated via post-translational processing of glucagon and is cosecreted with GLP-1, oxyntomodulin, and glicentin in a 1:1:1 ratio (17,18). Once GLP-2 has been released, its effects are mediated through the specific binding of the hormone to its receptor, GLP-2R which localizes to enteric neurons and endocrine cells (19,20). GLP-2 secretion is regulated by nutritional, hormonal, and neural factors and it has been suggested that many of its actions are exerted indirectly through secondary mediators such as growth factors from enteroendocrine, neural, and lamina propria cells. Some of its actions include upregulating motility, nutrient absorption, and cell proliferation while reducing epithelial permeability and apoptosis in the gut (18,21,22). Postprandial GLP-2 secretion is regulated in a complex manner consisting of both direct (via nutrients) and indirect (via endocrine and/or neural) pathways (17). Nutrient-stimulated GLP-2 release has been found to result in a 2- to 5-fold increase in plasma levels and as such can easily be detected in fed samples (23).

We have recently shown that low circulating concentrations of GLP-2 were associated with high levels of bacterial translocation, suggesting that impaired secretion may contribute to mucosal dysfunction during diarrhoea infection (24). The study set out to investigate what changes, if any occur in the levels of GLP-2 in children with diarrhoea and malnutrition, and whether GLP-2 release has any link to seasonality, as nutrient availability and transmission of enteropathogens are seasonal in Zambia.

## METHODS

### Study Participants

Five different groups of children were studied over the period May 2014 to April 2019. Approval to conduct these studies was obtained from the University of Zambia Biomedical Research Ethics Committee (UNZABREC).

### Group 1—Acute Diarrhoea

Twenty-eight children with acute diarrhoea, defined as 3 or more loose stools in a 24-hour period (median duration 3 days, interquartile range [IQR] 2–4) were recruited when they presented to St. Lawrence clinic (Misisi, Lusaka). The children were assessed by Integrated Management of Childhood Illness trained nurses and managed accordingly (25). Informed written consent was obtained from the caregiver, and a 2 mL blood sample collected at the time of recruitment. The children were then followed up and a convalescent blood sample obtained 3 weeks after the diarrhoea episode ended. UNZABREC approval (007-11-15, dated January 12, 2016).

### Group 2—Persistent Diarrhoea

These children were undergoing treatment in the malnutrition ward of the University Teaching Hospital, Lusaka, Zambia. They had PD (diarrhoea for >14 days, including skip days, median duration 21 days, IQR 14–28) and SAM (weight-for-length *z* score [WLZ/weight-for-height *z* score] <–3 standard deviation [SD] and/ or mid-upper arm circumference [MUAC] <115 mm and/or bilateral pitting oedema of nutritional origin). No pathogens were detected on first-line investigations (stool microscopy and culture). All the admitted children with SAM were managed according to World Health Organization (WHO) guidelines on inpatient management of SAM (26). A total of 21 children were recruited and informed written consent obtained from the caregiver. Blood samples were collected from the children. UNZABREC approval (006-01-13, dated April 15, 2013).

### Group 3—Controls for Groups 1 and 2

Seventy-five stunted but clinically healthy children were recruited from Misisi compound as contemporaneous controls for groups 1 and 2. They were children who were screened for malnutrition in a previously described nutrition outreach programme (27). Children from the community were screened for recent episodes of diarrhoea (within 1 month), nonsteroidal antiinflammatory drug use (within 1 month), antibiotic use (within 1 month), and excluded and treated if needed. These children were selected on the basis that they were not acutely malnourished, but 56% had stunting (against a background of 40% stunting across the whole country) (28).

### Group 4—Stunted Children

A total of 5660 children aged 0 to 18 months of age, resident in Misisi compound were screened for wasting(MUAC <125 mm and/ or weight-for-length/height *z* score of <–2 SD) and stunting (length-/ height-for-age *z* score of <–2 SD) to identify those with malnutrition or growth faltering. Two hundred ninety-seven of those identified, were then recruited into a study of biomarkers of environmental enteropathy in children, provided with nutritional rehabilitation and followed up to the age of 24 months or for a minimum of 12 months. Blood samples were collected at recruitment and at 3 months. For those children who did not respond to 3 months of nutritional rehabilitation, fed, and fasted samples were collected on consecutive days. Nonresponse was defined as lack of improvement in anthropometric parameters (weight-for length/length-for-age/weight-for-age *z* score >-2 SD), provided the child had not been ill in this period. Children were fasted for at least 4 hours before a sample was collected, as they were being prepared for endoscopy. Stool samples were also collected from the children as they came in for their first visit. 181 baseline blood samples were tested for GLP-2. UNZABREC approval (006-02-16, dated January 3, 2018).

### Group 5—Controls for Group 4

Contemporaneous controls (n = 46) were recruited for the stunted children to control for the time lapse between the studies, and temporal changes, seasonal changes, and an improvement in stunting from 40% to 35% (28). These children were well nourished (WLZ >-1) and resident in Misisi compound. Nineteen baseline blood samples were tested for GLP-2.

### Assessment of Nutritional Status

A paediatrician and trained study nurses carried out nutritional status assessment of all the participants based on WHO child growth standards (29). This was done using anthropometric measurements of MUAC, weight, and length/height. The measurements were done using a Mother-Child scale (SECA 874, Hamburg, Germany) for weight, Infantometers (SECA 416, Hamburg, Germany) for length, UNICEF height boards for height, and MUAC tapes. Raw data were entered into the WHO Anthro software v.3.2.2 (30) which then calculated the anthropometry scores.

### Measurement of Glucagon-like Peptide 2, Stool Markers, and Stool Pathogens

Plasma samples were tested for GLP-2 by Enzyme Linked Immunosorbent Assay (ELISA) (Millipore Corporation, St Charles, MO). The assay is a sandwich ELISA that only measures total GLP-2.

Faecal myeloperoxidase and calprotectin were measured as markers of inflammation in children with stunting. Myeloperoxidase was measured using the EDI Quantitative Faecal/Urine Myeloperoxidase ELISA Kit (Epitope Diagnostics Inc, San Diego, CA) and calprotectin using the IDK Calprotectin ELISA (Immundiagnostik AG, Bensheim, Germany). Both assays use the 2-site sandwich technique with 2 selected monoclonal antibodies binding to the protein of interest.

Stool pathogen analysis was done using the qualitative, multiplex polymerase chain reaction–based Luminex x-TAG gastrointestinal pathogen panel (Luminex Corporation, Austin, TX). This assay is able to simultaneously detect 15 enteric pathogens with a sensitivity of 94.3%, for 12 of the 15 pathogens, and a specificity of 98.5% across all 15 pathogens (31). Sample processing and assay conditions are described by Chisenga et al (32). All the assays were run according to the manufacturer’s instructions and more detail on the assays is provided in Supplemental Digital Content 1 (*http://links.lww.com/MPG/B783*).

### Data Regarding Environmental Exposures

Rainfall and temperature data for the period 2016 to 2018 were obtained from the Lusaka City Airport, Zambia Meteorological Office. Food Price Index (FPI) was obtained from the Jesuit Centre for Theological Reflection monthly survey (33). This survey details retail costs of basic food items of a family of 5. It is carried out in different towns and it averages the cost of food based on the prices gathered from different places within the town of interest. For the purposes of the article, the prices indicated were averaged from 2 markets in the vicinity of our study catchment area namely Chawama market and City market. This was to allow us to get more representative costs as averaging it across the costs of the entire town would not give a true picture.

### Data Analysis and Statistical Considerations

GLP-2 concentrations in serum were determined to be non-normally distributed using the Shapiro-Wilk test, so data are presented as median and IQR. The Kruskal-Wallis test was used for hypothesis testing. The Wilcoxon matched-pairs rank sum test was used to test paired samples, and the Friedman test for multiple comparisons. Spearman correlation was used to test for correlation between GLP-2 and stool inflammatory markers. A *P* value <0.05 was considered significant. Univariable and multivariable logistic regression analysis was carried out to determine if there was any association between GLP-2 concentrations and different variables and pathogens. Pathogen results with numbers <10 were not included in the final model. Missing data were treated as missing and no imputation was made. Data were analysed using GraphPad Prism 5, and STATA v.15.

## RESULTS

### Participant Characteristics

A total of 324 participants were studied over the course of 5 years. Groups 1 and 2 were comparable in sex and age but not in anthropometry measures (Table 1). The majority of the children from groups 1, 2, and 3 were stunted but those in group 2 were also wasted. The control group (Group 3) had not had diarrhoea in the month before recruitment, were slightly older with more female participants than the other 2 groups. The stunted children (group 4) were also older than their contemporaneous controls (group 5).

**TABLE 1:** Baseline characteristics in the 5 groups of children studied

	Group 1 (n = 28)	Group 2 (n = 21)	Group 3 (n = 75)*	Group 4 (n = 181)	Group 5 (n = 19)
Months of sample collection	January–February 2016	May 2014–February 2015	August–October 2014	October 2016–September 2018	April 2017–April 2019
Sex: male, n (%)	17 (66%)	14 (67%)	30 (40%)	107(59%)	6 (32%)
Age (median IQR)	16.5 (11.5, 22)	17 (12, 21)	20 (16, 29)	11 (7, 14)	4 (3, 5)
Weight for age (WAZ)	−1.78(−2.55, −0.69)	−3.48(−4.98, −2.7)	−1.21(−1.98, −0.52)	−2.57(−3.14, −2.21)	0.08 (−0.86, 0.55)
Weight for length (WLZ)	−0.92(−1.68, 0.38)	−2.80 (−4.11, −1.61)	−0.27(−1.07, 0.32)	−1.23(−1.86, −0.59)	0.73 (0.21, 1.49)
Length for age (LAZ)	−2.24(−2.69, −1.38)	−2.66 (−4.36, −1.72)	−2.25(−2.9, −1.18)	−2.99(−3.56, −2.57)	− 0.81(−1.01, −0.17)
Samples available during illness	Plasma (fed state) during illness and after convalescence	Plasma (fasted state) during illness	Plasma (healthy in fed state)	Serum (fed and fasted state); stool for pathogen analysis	Serum (fed only); stool for pathogen analysis

LAZ = length-for-age *z* score; WAZ = weight-for-age *z* score; WLZ = weight-for-length *z* score.

* Anthropometric data missing for 1 healthy control. Group 1—acute diarrhoea; group 2—persistent diarrhoea; group 3—controls for groups 1 and 2; group 4—stunted children; group 5—controls for group 4. Fed state assumed to be fed, not timed in relation to sample collection.

### Glucagon-like Peptide 2 Levels Rise in Acute Diarrhoea But Not Persistent Diarrhoea

**FIGURE 1:**
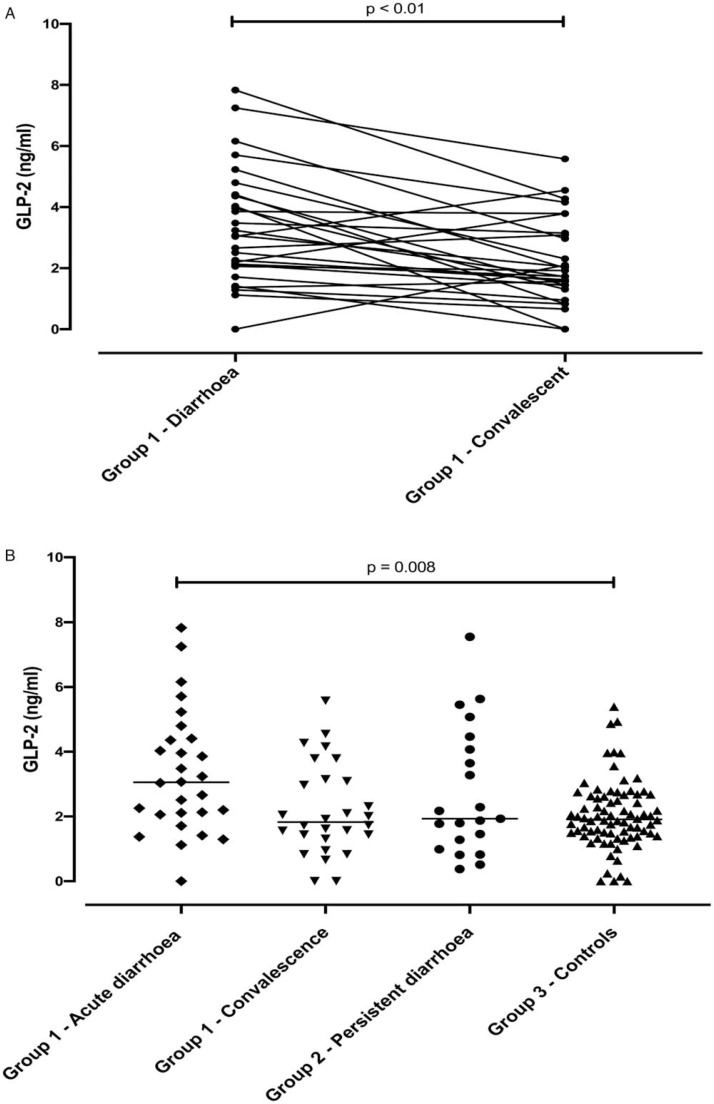
A, GLP-2 levels in children with acute diarrhoea during illness and 3 weeks after their last diarrhoea episode. B, GLP-2 levels in acute and persistent diarrhoea compared to controls. GLP-2 = glucagon-like peptide 2.

GLP 2 levels in children with acute diarrhoea (group 1), were elevated during illness and fell after convalescence (*P* = 0.001), in all except 5 children (Fig. 1A). Circulating GLP-2 concentrations after convalescence (median 1.83 ng/mL, IQR 1.38,3.12) were the same as in the controls (group 3) from the same community (median 1.87, IQR 1.40, 2.56), confirming that it was the samples taken during acute diarrhoea which were elevated (median 3.06 ng/mL, IQR 2.09, 4.39; Fig. 1B). Children with PD and SAM (group 2) had concentrations (median 1.93, IQR 1.28, 4.07) which were similar to control values (*P* = 0.33) (Fig. 1B).

### Glucagon-like Peptide 2 in Children With Stunting and Nonresponders

**FIGURE 2:**
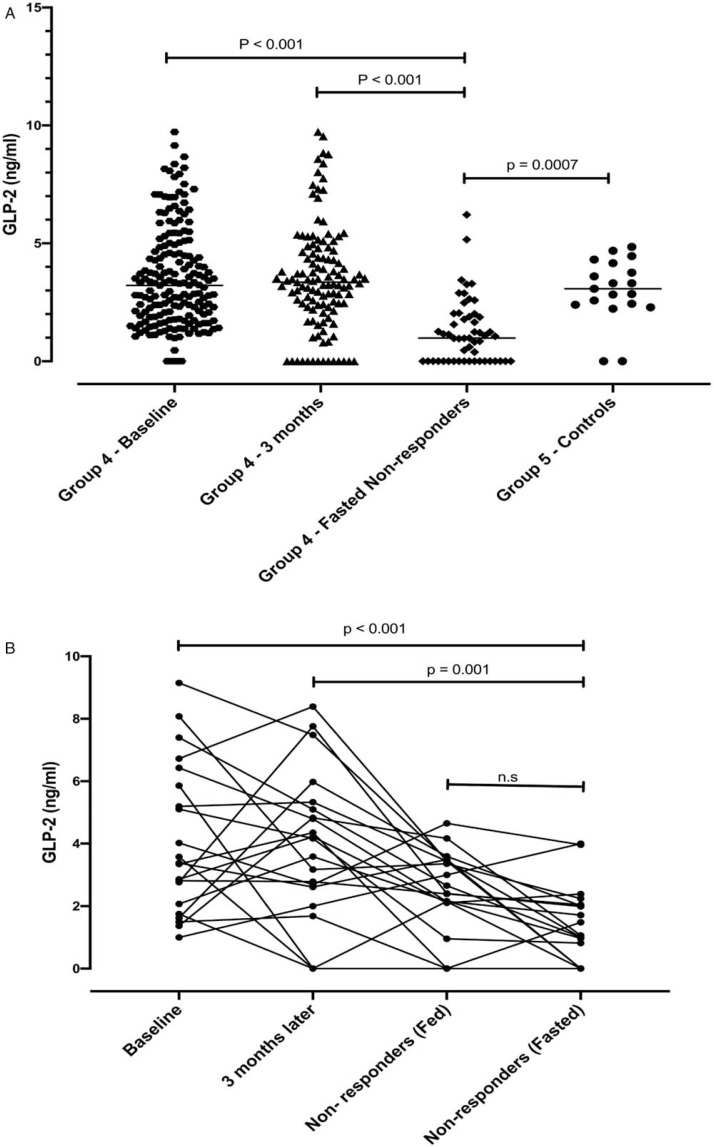
A, GLP-2 levels in stunted children at baseline, 3 months and before endoscopy (fasted nonresponders) compared to healthy controls from the same community. B, Repeated GLP-2 measurements in children designated nonresponders at baseline, 3 months, and in the fed and fasted state. Children were fed ad libitum. GLP-2 = Glucagon-like peptide 2.

In group 4, GLP-2 levels at recruitment (3.2, IQR 1.9, 4.9) did not differ from the 3-month visit (3.5, IQR 2.39, 4.83) or controls (3.1, IQR 2.39, 4.16), but were significantly lower in the fasted nonresponders (Fig. 2A). GLP-2 in the fasted state was 1.01 ng/mL (IQR 0, 2.01) and in the fed state was 2.40 ng/mL (IQR 2.10, 3.44). Fed and fasted values were both lower than baseline GLP-2 value (*P* < 0.001) suggesting that the fall in GLP-2 was not merely due to the fasted state (Fig. 2B).

### Glucagon-like Peptide 2 in Controls

In the control groups (groups 3 and 5), GLP-2 concentrations in plasma fell with age (ρ = −0.35; *P* = 0.001). Using an estimate of β from linear regression the change in GLP-2 over time would be a reduction of 0.04 ng/mL per month of increasing age. It is thus unlikely that the decline in GLP-2 observed in group 4 could be attributable to age.

### Stool Inflammatory Markers

In the baseline stool samples, median myeloperoxidase stool concentrations were at 102.51 ng/mL (IQR 40.37, 169.68) and calprotectin concentrations at 278.38 μg/g (IQR 164.73, 619.91). There was a negative correlation between GLP-2 and myeloperoxidase (ρ = −0.16, *P* = 0.02) and a positive correlation between GLP-2 and calprotectin (ρ = 0.18, *P* = 0.02).

### Glucagon-like Peptide 2 Shows Seasonal Pattern

Seasonal variation has been shown in the prevalence of undernutrition (34) and incidence of diarrhoea (35). Diarrhoeal pathogens have also been shown to vary seasonally, influenced by factors such as rainfall, temperature, and humidity (36). We set out to explore if there is any association between GLP-2 release and these factors that vary seasonally, that is, food availability, rainfall, temperature, and pathogens. The FPI, which covers the cost of food items such as maize meal, beans, kapenta, fish, and vegetables, constituents of a typical Zambian meal, was used as a proxy of food availability.

We found an association between GLP-2 and FPI, temperature, and rainfall. There was an increase in GLP-2 during the warmer months, and a decrease in GLP-2 when the cost of food was high, and during the rainy season. We also looked at whether there was any association between the different pathogens detected in stool and GLP-2 release as it has been documented that subclinical carriage of pathogens may result in enteropathy (37). Intestinal damage from enteropathy may result in a deficiency of hormonal response to nutrients. An association was found between GLP-2 and Shiga toxin-producing *Escherichia coli* (STEC). Multivariate regression was carried out to confirm these associations (Table 2).

**TABLE 2:** Univariable and multivariable logistic regression analysis showing the association between glucagon-like peptide 2 and different variables/pathogens

Variable	Univariable Analysis		Multivariable Analysis	
	Coefficient (β)	*P* (>0.05)	Coefficient (β)	*P* (>0.05)
Food Price Index	−0.002	0.06	−0.002	**0.05**
Minimum temperature	0.029	0.67	0.354	**<0.01**
Maximum temperature	0.177	**<0.01**		
Rainfall	−0.004	**<0.01**	−0.006	**<0.01**
*Adenovirus* (n = 20)	−0.642	0.25		
*Norovirus* (n = 104)	−0.457	0.22		
*Rotavirus* (n = 41)	−0.558	0.16		
*Campylobacter* (n = 78)	0.289	0.83		
Enterotoxigenic *Escherichia coli* (n = 117)	0.485	0.23		
Salmonella (n = 135)	−0.864	0.86		
Shiga toxin-producing *E coli* (n = 19)	−1.366	**0.02**	−1.409	**0.01**
*Shigella* (n = 109)	0.025	0.95		
*Cryptosporidium* spp (n = 43)	−0.318	0.43		
Giardia (n = 116)	0.138	0.73		

Results shown as β-coefficient and *P* value. Values presented in bold signify *P* ≤ 0.05.

## DISCUSSION

Taken together, our results suggest that GLP-2 levels are elevated in acute diarrhoea but not in PD and SAM. In children with acute diarrhoea, GLP-2 levels rose and then returned to background levels after recovery from infection. This suggests that in cases of acute diarrhoea, the intestinal response to infection includes an increase in the amount of circulating GLP-2 which would enhance repair of the damaged mucosal epithelium, thereby restoring epithelial integrity. A similar response has also been observed in children with intestinal failure (38) suggestive of the role GLP-2 plays in enhancing the adaptive response. In that study, mean fasted GLP-2 was 11.6 ng/mL in controls, and 19.9 ng/mL in children with intestinal failure. All the children in our study had considerably lower circulating GLP-2 concentrations than children in Finland (38) but as different ELISA kits were used, the difference noted could be due to methodological and biological factors. In PD, the reverse of what is occurring in acute diarrhoea was observed. It is plausible that in the children with PD, SAM plays a major role in the inability of the body to respond in a manner similar to that observed in the acute diarrhoea cases. An inability to produce GLP-2 may perpetuate the malnutrition-infection cycle by impairment of mucosal regeneration, and ultimately lead to death in children with SAM. In stunting, a progressive decline was noted in the children who did not respond to nutritional rehabilitation. It appears that malnutrition limits the ability of the gut to respond to intestinal damage. What is unknown is whether the enteroendocrine cells that sense these nutrients are impaired or have been lost in malnutrition.

One of the difficulties in interpreting these data is the lack of reference range for young children. Progressive increments in GLP-2 concentrations have been noted during early neonatal development and the weaning period (39). We included 2 groups of controls to provide a valid comparison. A further problem in children is difficulty in ascertaining the fed/fasted state. Although we attempted to be clear on this point, mothers often use breast-feeding to console their children and this may lead to misclassification. Composition of feeds also varies widely.

Intestinal GLP-2 release is nutrient stimulated, so nutrient availability partially determines GLP-2 release. We set out to examine whether there is any association between nutrient availability and GLP-2 release. We looked at seasonal parameters such as rainfall and temperature, which are known to affect food availability. We also took into consideration that the majority of Lusaka residents, very definitely those resident in our catchment area, are not farmers, so there is no direct link between produce and food availability. The cost of food and other essential household goods affects ones’ purchasing power, and consequently the amount and type of nutrition present in a home. We factored this into our analysis by use of the FPI, matching the cost of food at the time the sample was collected, with the amount of GLP-2 in the sample. Stool pathogens in samples collected over the same time frame were analysed to determine whether there was any association to GLP-2. All of the variables stated above were included in a multivariable logistic regression model with GLP-2 as the dependent variable. Rainfall, temperature, FPI, and STEC showed a significant association with GLP-2, with temperature having a positive correlation coefficient. According to WHO, the exposure routes for STEC infection include food, animal contact, human-to-human contact, water, and soil (40). Based on this, we can conclude that our findings of STEC in baseline stool samples are an indication of the levels of environmental contamination present in a population with high levels of environmental enteropathy. The levels of the stool markers found in our samples were indicative of the levels of gut inflammation present in our cohort. Both of these markers have been studied in relation to environmental enteropathy and malnutrition (41–45) with inconsistent results. We observed a negative correlation between GLP-2 and myeloperoxidase in our study, indicative of the high inflammation present in those with low GLP-2. The reverse was observed in calprotectin, but this result could have been confounded by the age and breast-feeding status of these children (46–48).

Our findings suggest that there is a link between seasonality and GLP-2 release, consistent with its role as a nutrient responsive hormone. Teduglutide, a GLP-2 analogue has been successfully used in the treatment of inflammatory bowel disease, and studies are now underway in severely malnourished children (49).
