# Supplementary material for: Glucagon-like Peptide 2 Concentrations Vary in Zambian Children During Diarrhoea, in Malnutrition and Seasonally
Source: J Pediatr Gastroenterol Nutr. 2020 Apr;70(4):513–20. doi: 10.1097/MPG.0000000000002633 (PMC7340486; doi:10.1097/MPG.0000000000002633)
Supplement: Supplementary file 1 [file JPGN-2020-MPG.0000000000002633-s1.docx]

*Suppl Content 1*

*Measurement of plasma GLP-2*

Blood samples were collected from the antecubital vein following local anaesthesia with 4% tetracaine gel, into BD Vacutainer plastic blood collection tubes (Becton Dickinson, Plymouth, UK), and spun down at 2700rpm for 15mins; plasma was then aliquoted in duplicate into 1.5ml tubes for storage at -80°C. Plasma samples were tested for total GLP-2 by Enzyme Linked Immunosorbent Assay (Millipore Corporation, St Charles Missouri, USA). The assay is a sandwich ELISA that measures the enzyme activity spectrophotometrically by the increased absorbance at 450nm-540nm after acidification of formed products. Briefly, 50ul of standards and samples were added in duplicate to a microtiter assay plate which contained 50ul of assay buffer. The plate was then incubated for 2 hours, washed and then incubated for another hour after addition of detection antibody. The plate was then washed a second time, before enzyme solution was added for 30minutes. 100uL of substrate solution was then added for 20mins before the reaction was stopped. The plate was read using a BioTek Plate Reader and analysed using Gen 5.1.10 software. The GLP-2 kit used does not differentiate between the inactive (1-33) and the active (3-33) peptide forms of GLP-2.

*Collection of stool and measurement of pathogens*

Baseline stool samples were collected as the children came for their first visit after recruitment. The collected samples were then transported to the laboratory within a 2-hour window, aliquoted into marked storage containers and stored at -80°C.

Stool pathogens were analysed using the qualitative, multiplex polymerase chain reaction (PCR) based Luminex x-TAG^®^gastrointestinal pathogen panel (Luminex Corporation, Austin, Texas, USA). This assay is able to simultaneously detect 15 enteric pathogens namely; Adenovirus 40/41, Campylobacter (C. jejuni, C. coli and C. lari only), Clostridium difficile (C. difficile) toxin A/B, Cryptosporidium(C. parvum and C. hominis only), Entamoeba histolytica (E. histolytica), Escherichia coli (E. coli) O157, Enterotoxigenic E. coli(ETEC) LT/ST, Giardia (G. lamblia/G. intestinalis/G. duodenalis only), Norovirus GI/GII, Rotavirus A, Salmonella, Shiga-like Toxin producing E. coli (STEC) stx 1/stx 2, Shigella (S. boydii, S. sonnei,S. flexneri and S. dysenteriae), Vibrio cholerae (V. cholerae) and Yersinia enterocolitica (Y. enterocolitica).

The assay was run according to the manufacturer’s instructions. The samples were thawed out and then homogenised by bead beating in SK38 soil grinding tubes (Cat KT03962-1-006.2, Bertin Corporation, USA). The homogenised sample was then incubated in a lysis buffer (NucliSENS^®^ easyMAG^®^ lysis buffer, BioMérieux, France) containing bacteriophage MS2 as an internal positive control, After lysis the sample was centrifuged and the supernatant collected was used to extract nucleic acids using the commercially available QIAamp MinElute Virus Spin kit(Qiagen, Germany). The nucleic acid extract was then used as a template in the x-TAG gastrointestinal panel PCR reaction mix.

*Measurement of Stool Myeloperoxidase and Calprotectin*.

Myeloperoxidase was measured using the EDI™ Quantitative Fecal/Urine Myeloperoxidase ELISA Kit (Epitope diagnostics, Inc. San Diego, USA). Briefly, 38mg of stool was weighed out and mixed with 1.5ml extraction buffer by vertexing. It was then let to sit for 30 mins before being centrifuged at 3000 x g for 5 mins. The supernatant was then transferred into a new tube for measurement of the fecal myeloperoxidase. The assay was run according to the manufacturers instruction with the absorbance read at 405nm with the reference filter at 630. The average absorbance of each pair of duplicate test results was then used to determine the myeloperoxidase present in each sample using a standard curve in ng/ml.

Calprotectin was measured using the IDK® Calprotectin ELISA (Immundiagnostik AG, Bensheim, Germany).15mg of sample was added to 1.5ml extraction buffer, allowed to stand for 10mins to let the sediment settle and then the supernatant was collected. The supernatant was then diluted 25-fold in dilution buffer before it was assayed. The manufacturer’s instructions were followed, and the absorbance was read at 450nm with a reference filter of 630. The Calprotectin values were determined using a standard curve in ug/g.
